# Self-Assembly of Short Elastin-like Amphiphilic Peptides: Effects of Temperature, Molecular Hydrophobicity and Charge Distribution

**DOI:** 10.3390/molecules24010202

**Published:** 2019-01-08

**Authors:** Meiwen Cao, Yang Shen, Yu Wang, Xiaoling Wang, Dongxiang Li

**Affiliations:** 1 State Key Laboratory of Heavy Oil Processing and Centre for Bioengineering and Biotechnology, China University of Petroleum (East China), 66 Changjiang West Road, Qingdao Economic Development Zone, Qingdao 266580, China; shenyang52633@163.com (Y.S.); m18254289278@163.com (Y.W.); 2 Personnel Department and School of Blue Economy Engineering, Qingdao Vocational and Technical College, Qingdao Economic and Technological Development Zone, Qingdao 266555, China; wangxl@qtc.edu.cn; 3 Shandong Key Laboratory of Biochemical Analysis, College of Chemistry and Molecular Engineering, Qingdao University of Science and Technology, Qingdao 266042, China; lidx@qust.edu.cn

**Keywords:** amphiphilic peptides, elastin-like peptides, elastomeric β-turn units, temperature-sensitivity, self-assembly

## Abstract

A novel type of self-assembling peptides has been developed by introducing the basic elastomeric β-turn units of elastin protein into the amphiphilic peptide molecules. The self-assembly behaviors of such peptides are affected by the overall molecular hydrophobicity, charge distribution and temperature. The molecules with higher hydrophobicity exhibit better self-assembling capability to form long fibrillar nanostructures. For some peptides, the temperature increase can not only promote the self-assembly process but also change the self-assembly routes. The self-assembly of the peptides with two charges centralized on one terminal show higher dependence on temperature than the peptides with two charges distributed separately on the two terminals. The study probes into the self-assembly behaviors of short elastin-like peptides and is of great help for developing novel self-assembling peptides with thermo sensitivity.

## 1. Introduction

Elastin is an insoluble extracellular-matrix protein that imparts elasticity to organs and tissues [1,2,3]. Tropoelastin is the soluble precursor of elastin. It can form elastic fibers, which then cross-link to produce elastin [4]. Tropoelastin contains two alternating domains, one hydrophilic domain rich in alanine (A) and lysine (K) residues and one hydrophobic domain rich glycine (G), A, valine (V), and proline (P) residues [5]. The hydrophilic domain provides cross-linking sites that can form covalent bonds to connect the polypeptide chains. The hydrophobic domain usually has repeated β-turn peptide sequences such as VPGG, VPGVG and APGVGV [1,6,7,8,9,10,11], which can introduce specific viscoelasticity and temperature-sensitivity to the molecule [8,9].

Inspired by elastin, elastin-like polypeptides (ELPs) that are composed of the characteristic elastomeric units of tropoelastin have been developed as the artificial biopolymers [2,12]. ELPs usually show temperature-sensitive folding behaviors and they can give conformational changes upon heating [13,14]. It has been demonstrated that the VPGVG-based ELPs collapse from an extended chain to a type II β-turn conformation around the Pro-Gly pair with temperature increase [15,16,17,18]. Such a phenomenon is usually termed inverse temperature transition (ITT) [13,19,20], which is suggested to be driven by dehydration of the hydrophobic valine side chains [21]. Very interestingly, short elastin-derived peptides with limited number (usually 1–5) of the repeating β-turn units have been shown to have similar temperature-sensitive conformational changes as ELPs [20,21,22,23,24,25,26,27,28,29]. Reiersen et al. showed that a single VPGVG unit can act as a thermodynamically independent unit to undergo the temperature-induced extended‒β-turn transition [21]. The fact established that the temperature-driven conformational change is an intrinsic property of the individual β-turn sequence. Moreover, it has been found that the ITT temperature of an elastin-like peptide can be tailored through several aspects, such as adjusting the chain length, replacing residues in the repeating unit, varying groups and charges at the termini and so on [22,28,30,31]. These findings all serve as foundations for designing novel elastin-based short peptides that have temperature-responsive self-assembling properties [29,32,33,34,35].

Short amphiphilic peptides are one important category of self-assembling peptides that can form distinct nanostructures [36,37]. By having specific mechanistic property and high biocompatibility, these nanostructures show potential applications in vast areas including templates for nanostructure construction [38], scaffolds for tissue engineering [39,40], carriers for medicine delivery [41,42,43], and biological surface engineering [44,45]. It is of great interest to see whether we can develop temperature-responsive self-assembly molecules by involving the elastin β-turn units into the amphiphilic peptides. In the present study, a series of elastin-based peptides have been designed by introducing one or two VPGVG units into the amphiphilic peptide molecule, as shown in Table 1. The repeated I sequence (III) was designed to give hydrophobicity and self-assembling capability [46,47]. Two K residues were introduced to give positive charges. Their different positions in the molecules can provide evaluation of the effect of charge distribution on molecular self-assembly. For comparison, two molecules with only one VPGVG unit but without III segment were also synthesized. The study investigates the effects of molecular hydrophobicity, charge distribution and temperature on the self-assembly behaviors of the designed peptides. It bears great significance for the rational design of temperature-sensitive self-assembling peptides.

## 2. Experimental

### 2.1. Materials and Solution Preparation

All of the peptide molecules were obtained from GL Biochem (Shanghai) Ltd. (Shanghai, China). The purity was ≥96%. All solutions were prepared in water with a resistivity of 18 MΩ cm that was obtained from a Millipore purification system (Millipore Corp, MA, USA). After adding water into the vial with peptide powder, the sample was treated with bath sonication for 15 min. The solution pH was 4.5 ± 0.3 due to presence of adventitious trifluoroacetic acid in the sample. The fresh solutions were incubated at either 20 °C or 80 °C (sealed tightly and incubated in an air oven). All solutions were aged for at least one day for reaching equilibrium before equipment characterization. 

### 2.2. Fluorescence Measurement

A Fluoro Max-P spectrophotometer (JOBIN YVON, Paris, France) was used for fluorescence measurements at room temperature. A 0.5 cm quartz cuvette was used. The critical aggregation concentrations (CACs) of each peptide were determined by using pyrene as a fluorescence probe. The emission spectra of pyrene in the 350‒500 nm range were collected with excitation at 335 nm. The pyrene polarity index I_1_/I_3_, that is, the ratio between the fluorescence intensities of peaks I and III, was calculated and plotted as a function of concentration. 

### 2.3. Atomic Force Microscopy (AFM)

AFM measurements were carried out on a Multimode Nanoscope IVa AFM (Digital Instruments, Santa Barbara, CA, USA) under ambient conditions. TESP silicon probes with a nominal spring constant of 42 N/m (Veeco, Santa Barbara, CA, USA) were used for collecting the tapping mode morphologies. The experimental parameters were as follows, scan speed: 1.0–1.8 Hz, tip resonance frequency: 230–300 kHz, and drive amplitude: 20–100 mV. For sample preparation, a drop of 10–15 μL peptide solution was deposited onto a freshly cleaved mica surface. After adsorption for 5–30 s the sample was dried under a nitrogen stream. The sample was immediately subjected to AFM imaging. Analysis of the AFM results was carried out using the vendor-supplied software version V530r3sr3 (Veeco, Santa Barbara, CA, USA). In experiments, different nitrogen flow speeds were applied for sample preparation and no difference in the sample morphologies were found. This excluded the impacts of solvent evaporation and solvent gradient forces on the peptide self-assembled structures.

### 2.4. Transmission Electron Microscopy (TEM)

TEM measurements were performed on a JEM-2100UHR electron microscope (JEOL, Tokyo, Japan) at 200 kV. TEM samples were prepared using the negative staining method. Firstly, a carbon Formvar-coated copper grid was placed on a drop of peptide solution. After adsorption for about 8 min, the grid was removed from the solution and then placed on a drop of uranyl acetate solution (2% *w*/*v*) for negative staining. The samples were subjected to TEM characterization immediately after preparation.

### 2.5. Circular Dichroism (CD)

A MOS-450/AF-CD spectrophotometer (BioLogic, Paris, France) was used to collect the CD spectra. A 1.0 mm quartz cell was used. Scans were collected by taking points at 0.5 nm and an integration time of 0.5 s. Three repeats were performed for each curve and the averaged results were given. Spectra smoothing and noise reduction were carried out using the vendor-supplied software.

### 2.6. Fourier Transform Infrared Spectroscopy (FTIR)

Infrared spectra were recorded on a Nicolet 6700 FT-IR spectrometer (Thermo Fisher Scientific, Waltham, MA, USA). The peptide solution was firstly deposited on a CaF_2_ plate and dried under vacuum. The peptide deposits were then resuspended with D_2_O and subsequently dried to form thin films. The resuspension procedure was repeated three times to ensure maximal hydrogen-to-deuterium exchange. The spectra were taken using a 4 cm^−1^ resolution and averaging of 128 scans.

## 3. Results and Discussion

### 3.1. Determination of CACs

The six peptides vary in the overall molecular hydrophobicity by having or having not the III segment and the number of the VPGVG unit. Moreover, all of the peptides are blocked at their C and N terminals, each one carrying two positive charges from the two K residues at the studied pH of 4.5 ± 0.3. The two charges either centralize on one terminal or distribute separately on the two terminals. Therefore, we can probe into the effects of molecular hydrophobicity and charge distribution on the peptide self-assembly behaviors.

**Figure 1:**
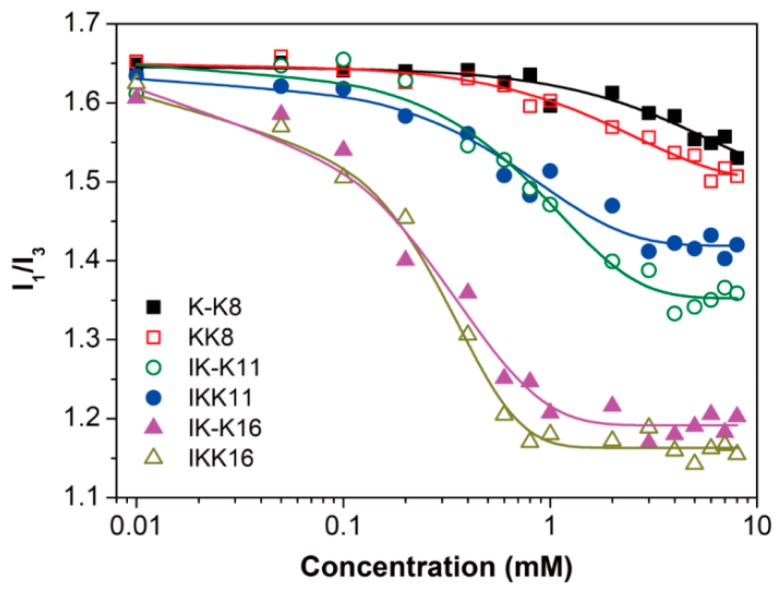
Variation of the pyrene polarity ratio I_1_/I_3_ with the peptide concentration.

First, the CAC of each peptide was determined using the pyrene fluorescence probe method [48,49]. The results are shown in Figure 1, where the pyrene polarity ratio (I_1_/I_3_) was plotted as a function of peptide concentration. The CACs were determined from intersection of the extrapolations of the decreasing part of each curve and the nearly horizontal part at relatively higher concentrations [50]. The results are listed in Table 2. For K-K8 and KK8, the two 8-mer molecules with one VPGVG unit but no III segment, they all gave CACs of larger than 8.0 mM. For IK-K11 and IKK11, the two 11-mer molecules with one VPGVG unit linked to the III segment, they gave moderate CACs of around 2.65 mM and 2.20 mM, respectively. While for IK-K16 and IKK16, the two 16-mer molecules with two VPGVG units, they gave much smaller CACs of about 0.94 mM and 0.74 mM, respectively. The results clearly show that the CACs depend greatly on the molecular hydrophobicity, while the charge distribution has limited effect on the CACs of the peptides with the same length.

### 3.2. Morphologies of the Self-Assembled Structures

**Figure 2:**
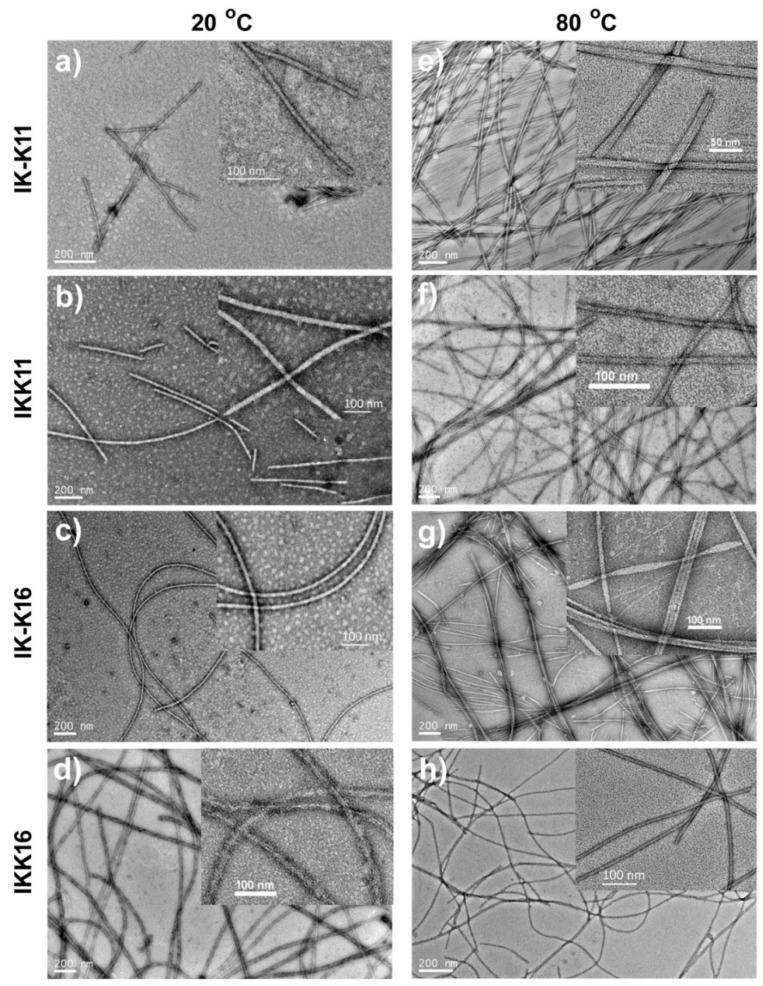
TEM morphologies show the self-assembled structures of different peptides at concentration of 4.0 mM and temperature of either 20 °C or 80 °C.

The self-assembled structures of each peptide at 4.0 mM and varied temperature were further investigated by TEM and AFM. Since the studied concentration was below the CACs of the 8-mer peptides, it is reasonable that K-K8 and KK8 both gave amorphous aggregates (Figure S1, Supporting Information). However, for the 11-mer and 16-mer peptides, they all self-assembled into distinct nanostructures whose morphologies showed dependence on temperature, as shown in Figure 2. IK-K11 formed short smooth fibrils with limited length of <600 nm and a relatively uniform diameter of 11 ± 2.5 nm at 20 °C. However, it gave long fibrils with length of several micrometers and diameter of a quite large range of 15 ± 8 nm at 80 °C. The inset magnified TEM image in Figure 2e gave several fibrils with distinct substructures. Clearly the fibrils are comprised of thinner primary fibers with diameter of ~4.0 nm. The number of such primary fibers varied for each fibril, resulting in broad distribution of the fibril diameters. IKK11 also produced fibrils with limited length of <1200 nm at 20 °C, which had smooth surface. Their diameters were quite large to be 20 ± 3.5 nm. The self-assembled nanostructures also turned into long fibrils of micrometers long at 80 °C, whilst the diameters became significantly smaller to be 12.0 ± 2.0 nm. The large difference in diameters indicates that the fibrils formed at 80 °C were not from elongation of the fibrils formed at 20 °C. They might come from different self-assembling routes. The two 16-mer peptides exhibited quite different self-assembly behaviors. IK-K16 formed long smooth fibrils of micrometers in length and 13.5 ± 2.5 nm in diameters at 20 °C. However, it produced two kinds of aggregates 80 °C, that is, the separate long fibrils and the aggregated fibril bundles. These fibrils had diameters of 14.0 ± 2.0 nm, approximately the same as the fibrils formed at 20 °C, indicating they were from the same assembly route. IKK16 also produced fibrils of micrometers long at 20 °C. However, the fibrils showed rough surfaces and their diameters were quite large to be 20.0 ± 5.5 nm. Interestingly, the fibrils produced by IKK16 at 80 °C gave smooth surfaces and significantly smaller diameters of 11.0 ± 1.0 nm. Similar to the case of IKK11, here the differences in surface roughness and diameters also indicate the fibrils formed at 20 °C and 80 °C might come from different self-assembling routes. The self-assembled nanostructures of each peptide were also imaged by AFM. The results (Figure S2, Supporting Information) were well consistent with the TEM results.

### 3.3. Secondary Structures at Varied Temperatures

**Figure 3:**
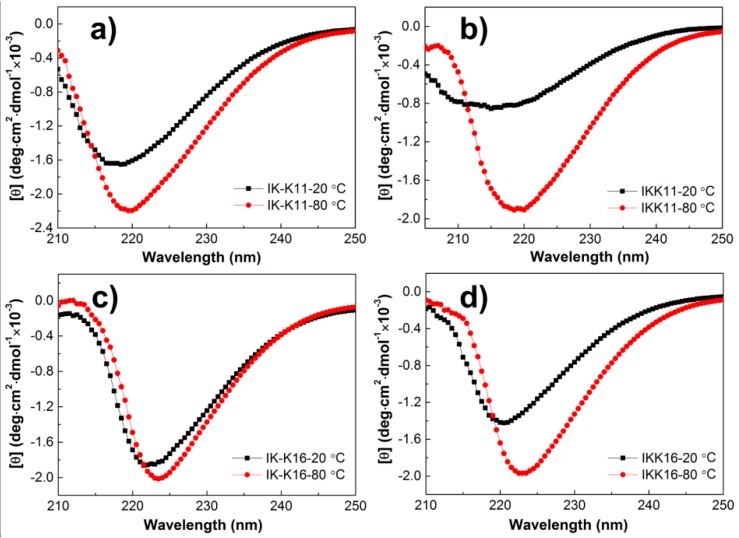
The CD spectra of different peptide solutions at concentration of 4.0 mM and temperature of either 20 °C or 80 °C.

**Figure 4:**
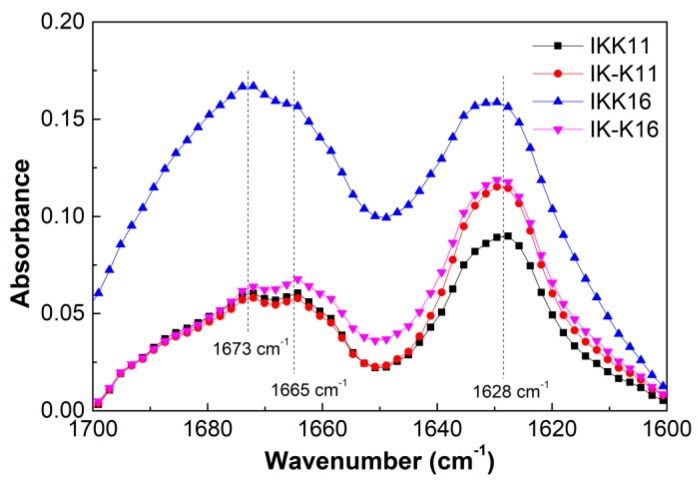
The amide I region of the FTIR spectra of different peptide solutions at concentration of 4.0 mM and temperature of 20 °C.

To further probe into the effect of temperature on peptide self-assembly, CD spectra of the peptide solutions were first obtained to infer the secondary structural change. Figure 3 presents the CD spectra of the peptide solutions of 4.0 mM at different temperatures. The 11-mer and 16-mer peptides all gave negative bands with varied peak positions at 20 °C, that is, 218 nm for IK-K11, a broad band between 212 and 220 nm for IKK11, 222 nm for IK-K16, and 221 nm for IKK16, respectively. These negative bands near 220 nm can be ascribed to the n–π* transition, indicating the β-sheet secondary structures [51], while shift in the peak position should be associated with distortion of β-sheets [52]. In fact, such CD spectra are typical for short elastin-like peptides [18,21,30]. FTIR measurements were further performed to confirm the secondary structures. The spectra (Figure S3) all showed characteristic peptide bands, that is, amide I, amide II and amide III bands. Specifically, the amide I region (Figure 4) for each peptide all gave a prominent band at ~1630 cm^−1^ and two shoulders at ~1665 and 1673 cm^−1^. The peak at 1630 cm^−1^ can be ascribed to β-sheet secondary structure [53,54], being in consistence with the CD results. The peak at 1673 cm^−1^ is suggested to indicate an antiparallel *β*-strand arrangement [49,55].

Comparing to the CD spectra at 20 °C, the spectra for each peptide at 80 °C all gave an obvious increase in intensity as well as a slight red shift to larger wavelength. The intensity increase indicated that more β-sheets were formed in the solutions at higher temperature. It can be rationalized that the temperature increase promoted the self-assembly process by providing more energy. The shifts in peak wavelength indicated that the temperature increase also induced change in molecular conformations and arrangements, resulting in distortion of the β-sheets [52]. Moreover, the peptides with two positive charges on one terminal, that is, IKK11 and IKK16, gave larger increase in peak intensity than their counterparts of IK-K11 and IK-K16 with two positive charges separated on the two terminals. Taking also the morphological transitions of the self-assembled nanostructures into consideration, we deduce that the temperature increase not only promoted the self-assembly process but also induced change of the self-assembly routes of IKK11 and IKK16.

Previous studies demonstrated that the shorter elastin-like peptides were less-disordered than the longer peptides at low temperatures [21,30]. However, in current systems, IKK16 gave more negative peak than IKK11 and IK-K16 gave more negative peak than IK-K11 at 20 °C, indicating that the longer peptides produced nanostructures with more ordered molecular arrangements. The phenomenon was proposed to be resulted from introduction of the highly hydrophobic III segment into the peptides, which greatly tuned the self-assembly behaviors. 

**Figure 5:**
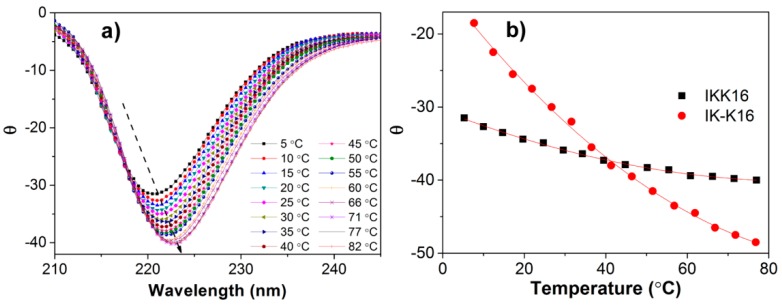
(**a**) The CD spectra of IKK16 solution (4.0 mM) at different temperatures. (**b**) Variation of the intensity of the negative peak at 220–225 nm as a function of temperature.

For further probing into the effect of charge distribution on peptide self-assembly, we recorded the CD spectra of IK-K16 and IKK16 at varied temperatures during a gradual temperature increasing course. The results are shown in Figure 5. The gradual shift of the negative β-sheet peak towards larger wavelength with temperature increase can be clearly seen in Figure 5a. Since the peak intensity reflects the β-sheet content, dependence of the self-assembly extent on temperature was shown in Figure 5b by plotting the peak intensity as a function of temperature. Comparing to IK-K16, IKK16 gave a more sharp decrease in θ value (increase in β-sheet content) with temperature increase. It indicated that the self-assembly of IKK16 (with two charges centralized on one terminal) showed higher dependence on temperature than IK-K16 (with two charges distributed separately on the two terminals).

### 3.4. Effects of Molecular Hydrophobicity, Charge Distribution and Temperature on Peptide Self-assembly

Several interesting experimental results can be concluded, which may find interpretation from the aspects of molecular hydrophobicity, charge distribution and temperature. First, the CACs decrease in the order of the 8-mer peptides, the 11-mer peptides and the 16-mer peptides, that is, the self-assembling capability increases with increasing chain length. This can be interpreted from the aspect of molecular hydrophobicity. As known, hydrophobic interaction is one of the main driving forces for peptide self-assembly. Here, the longer peptides have higher molecular hydrophobicity and reasonably give higher level of intermolecular hydrophobic interaction during self-assembly. This facilitates molecular aggregation at lower concentrations, that is, to give smaller CACs.

Then, there are two interesting facts about the dimensions of the self-assembled structures at 20 °C. One is that the two 11-mer peptides produced short fibrils with limited length while the two 16-mers produced long fibrils that seemed to have infinite length. The other is that IK-K11 and IK-K16, the peptides with the positive charges separated on the two terminals, gave smaller fibril diameters than their counterparts of IKK11 and IKK16, whose charges are centralized on one terminal. For all of these four peptides, the self-assembly driving forces should include hydrophobic interaction, hydrogen bonding and electrostatic interaction. These forces regulated the growth of the nanostructures along each dimension, and the interplay and balance determined the final self-assembled nanostructures [43,49]. First, the axial growth is driven by hydrogen bonding between β-strands in a directional way. Here it is apparent that the axial hydrogen bonding formation had excellent continuity in the cases of the 16-mer peptides while it was easily disrupted in the cases of the 11-mers. Since the four peptides all have two positive charges, electrostatic interaction should be approximately the same for them. However, as discussed in the above text, the 11-mers and the 16-mers had different levels of hydrophobic interaction due to their varied molecular hydrophobicity. The 11-mers had relatively weaker hydrophobic interaction, which might not be strong enough to afford the conformational distortion when hydrogen bonding was consecutively formed along the long axis, resulting in restricted fibril length. While for the 16-mer peptides, hydrophobic interaction was greatly enhanced, which could stabilize the aggregates and facilitate the axial hydrogen bonding for producing long fibrils. Then, for the fibril diameter, it should be mainly determined by the number of β-sheets that stacked along the zippering axis. The fibrils of these four peptides all had positive charges distributed on their surface, as evidenced by the positive zeta potentials of the peptide solutions (data not shown here). For IK-K11 and IK-K16, one positively charged Lys residue is connected to the III segment that formed the hydrophobic inner core during self-assembly. To expose this charge outward the peptide molecule should take a highly folded conformation. Such a conformation might be unfavorable for stable and continuous stacking of β-sheets along the zippering axis and therefore resulted in relatively smaller diameters.

For the effect of temperature, we can discuss from the following aspects. First, the temperature increase provided more energy to promote peptide self-assembly. This can be confirmed by the facts of both the growth of the fibril length and the increase in β-sheet content. Second, the temperature increase would change the molecular conformation by inducing the temperature-responsive VPGVG unit to fold. Such a conformational change might have case-dependent outcomes. One is that it enabled higher level of lateral association of the fibrils, as in the case of IK-K16. It might also change the self-assembling routes by passing through other energy barriers, as very likely in the cases of IKK11 and IKK16.

## 4. Conclusions

A series of short self-assembling peptides, that is, the 8-mer peptides of K-K8 and KK8, the 11-mer peptides of K-K11 and KK11, and the 16-mer peptides of K-K16 and KK16, have been designed and synthesized by introducing one or two elastomeric VPGVG units into the amphiphilic peptide molecules. The effects of molecular hydrophobicity, charge distribution and temperature on the peptide self-assembly behaviors were investigated. The results showed that the two 8-mer molecules with only one VPGVG unit but non III segment had quite low self-assembling ability and cannot form ordered structures at concentrations smaller than 8.0 mM. At lower temperature of 20 °C, the two 11-mer peptides self-assembled into short fibrils, whilst the two 16-mer peptides formed long fibrils. At higher temperature of 80 °C, both the 11-mer and 16-mer peptides produced long fibrils and/or fibril bundles. For the two molecules with centralized charge distribution, that is, IKK11 and IKK16, the temperature increase can promote their self-assembly process as well as tune the self-assembly routes. Moreover, comparing to IK-K16 the self-assembly of IKK16 showed much higher dependence on temperature. The study demonstrates the exciting possibility of developing temperature-sensitive short self-assembling peptides by introducing the basic elastin β-turn units into the molecules, which is believed to be of great help for the construction of peptidic materials with functionality.

## Figures and Tables

**Table 1:** Sequences of the designed short elastin-based peptides.

Peptide Sequence	Abbreviation
Ac-KGVPGVGK-NH_2_	K-K8
Ac-GVPGVGKK-NH_2_	KK8
Ac-IIIKGVPGVGK-NH_2_	IK-K11
Ac-IIIGVPGVGKK-NH_2_	IKK11
Ac-IIIKGVPGVGVPGVGK-NH_2_	IK-K16
Ac-IIIGVPGVGVPGVGKK-NH_2_	IKK16

**Table 2:** List of the CACs, the self-assembled structures and the corresponding size parameters of all peptides.

Peptide	Temperature	CAC (mM)	Self-Assembled Structures	Size Parameters (nm)	
				Length	Diameter
K-K8	20 °C	>8.0	amorphous aggregates	—	—
	80 °C	—	amorphous aggregates	—	—
KK8	20 °C	>8.0	amorphous aggregates	—	—
	80 °C	—	amorphous aggregates	—	—
IK-K11	20 °C	2.65 ± 0.26	short fibrils	<600	11.0 ± 2.5
	80 °C	—	long smooth fibrils	>1000	15.0 ± 8.0
IKK11	20 °C	2.20 ± 0.17	short fibrils	<1200	20.0 ± 3.5
	80 °C	—	long smooth fibrils	>1000	12.0 ± 2.0
IK-K16	20 °C	0.94 ± 0.11	long smooth fibrils	>1000	13.5 ± 2.5
	80 °C	—	long smooth fibrils & fibril bundles	>1000	14.0 ± 2.0
IKK16	20 °C	0.74 ± 0.08	long rough fibrils	>1000	20.0 ± 5.5
	80 °C	—	long smooth fibrils	>2000	11.0 ± 1.0

## Supporting Information

The following are available online. TEM and AFM morphologies of the peptide self-assembled nanostructures.
