# Supplementary material for: Self-Assembly of Short Elastin-like Amphiphilic Peptides: Effects of Temperature, Molecular Hydrophobicity and Charge Distribution
Source: Molecules. 2019 Jan 8;24(1):202. doi: 10.3390/molecules24010202 (PMC6337584; doi:10.3390/molecules24010202)
Supplement: Supplementary file 1 [file molecules-24-00202-s001.pdf]

**Supporting Information for**

# Self-Assembly of Short Elastin-like Amphiphilic Peptides: Effects of Temperature, Molecular Hydrophobicity and Charge Distribution

Meiwen Cao,[*,1] Yang Shen,[1] Yu Wang,[1] Xiaoling Wang,[2] Dongxiang Li[3]

*[1] State Key Laboratory of Heavy Oil Processing and Centre for Bioengineering and Biotechnology, China University of Petroleum (East China), 66 Changjiang West Road, Qingdao Economic Development Zone, Qingdao 266555, China*

*[2] Qingdao Vocational and Technical College, Qingdao Economic and Technological Development Zone, Qingdao, P. R. China*

*[3] Shandong Key Laboratory of Biochemical Analysis; College of Chemistry and Molecular Engineering, Qingdao University of Science and Technology, Qingdao 266042, PR China*

**Corresponding authors: phone & fax: 86-532-86983455, email:* ***mwcao@upc.edu.cn***

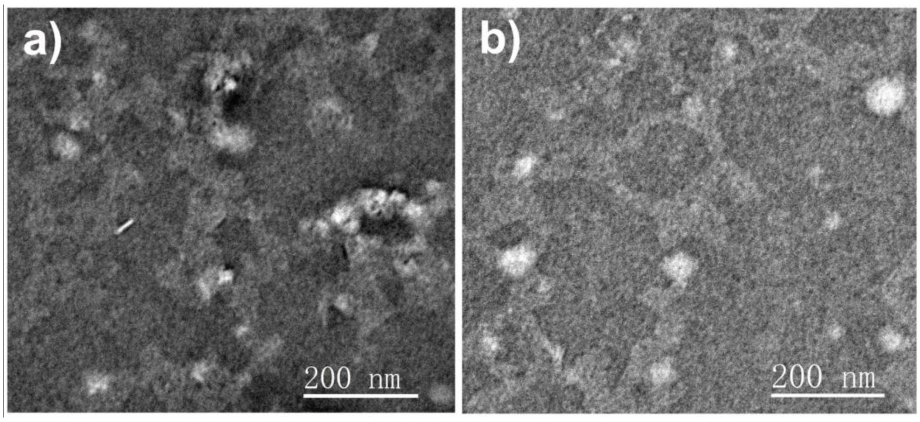


**Figure S1.** TEM morphologies show the aggregate structures of peptides IK-K8 (left) and IKK8 (right) at concentration of 4.0 mM and temperature of either 20 °C.

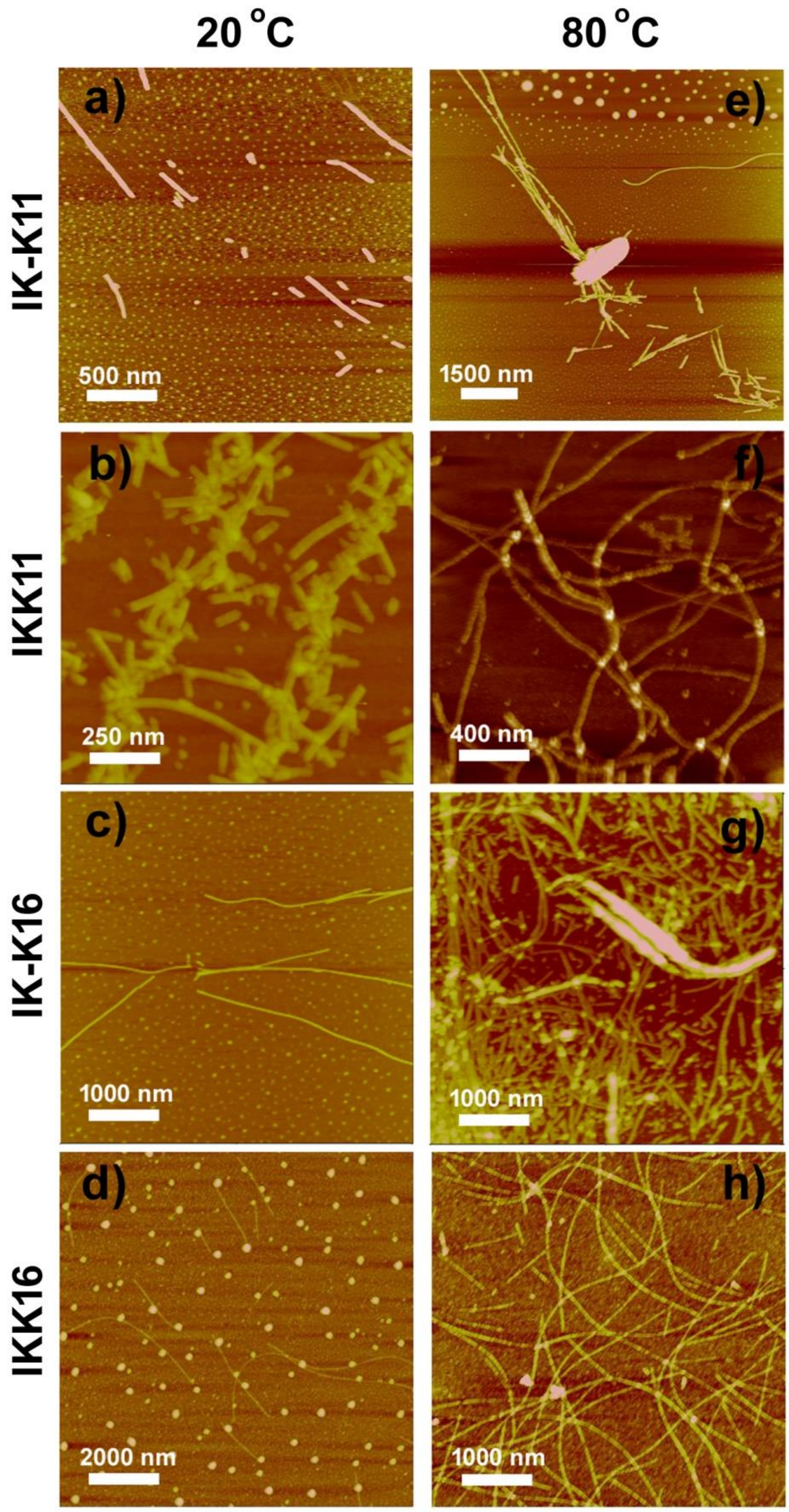


**Figure S2.** AFM morphologies show the self-assembled structures of different peptides at concentration of 4.0 mM and temperature of either 20 °C or 80 °C.

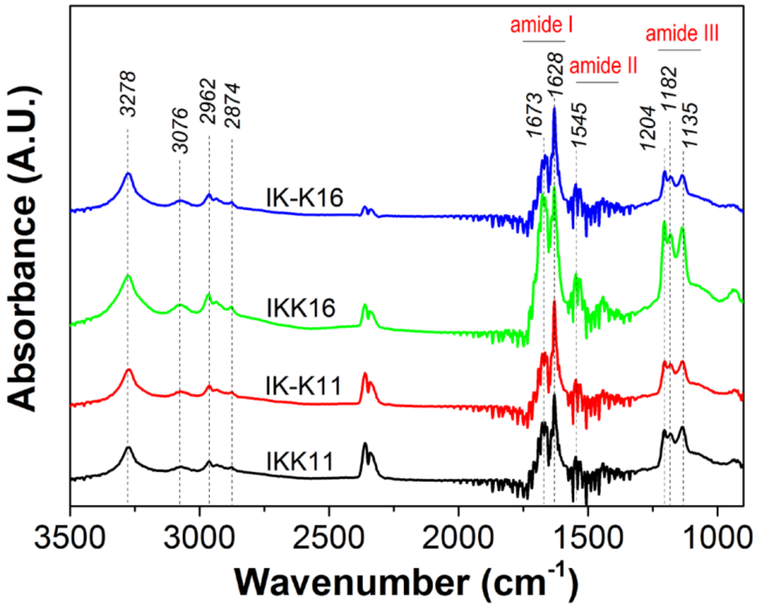


**Figure S3.** The FTIR spectra of different peptide solutions at concentration of 4.0 mM and temperature of 20 °C.
